# To the Editors of the Medical and Physical Journal

**Published:** 1803-05-01

**Authors:** A. Carlisle

**Affiliations:** Soho Square


### *To the Editors of the Medical and Physical Journal.*

Gentlemen,

THE letter from Mr. S. Cooper in your last Journal, demands my thanks for his ready explanation.

It was never my design to assume any merit for priority as the inventor of a cure for Herniæ. I had consulted all the orthodox authorities on the subject of such operations before I ventured to form a plan of my own, founded on the modern improvements in surgery. These thoughts were eventually laid before my Colleagues at the Westminster Hospital, on an occasion which seemed to warrant the enterprise.

A bricklayer's labourer under twenty years of age, applied to me for the means of procuring a truss; he had a Scrotal Hernia, descending through a widely dilated abdominal ring, and the nature of his work had rendered it difficult for him to keep a sufficient pad firmly over the orifice. The moral considerations of this individual case, induced me to propose an operation to him for a radical cure, and he consented to abide the event. My colleagues were of opinion, "That the danger to life was inconsiderable, but that the chance of the intended success was not great." I willingly risked my professional character on the latter point, because I had reason to be assured in my own judgment, founded on the peculiar precautions in my intended operation, that the success would be nearly certain. This man, however, left the hospital clandestinely, after having been cured of the venereal disease.

As Mr. Cooper's first proposal contained many of the very quotations in favour of an operation for the radical cure of bubonocele, which I had publicly urged, and as only the initials of his name appeared to that proposal, it looked as if some unknown person had usurped that pretension without any of the merit which I now acknowledge is due to Mr. Cooper.

The particulars of my designs for conducting this operation are, at present, unknown to any other person; and it may be sufficient for the present purpose to say, that I have deliberately weighed the moral and professional considerations which attach to this subject, and having compared these with my experience in similar operations, I have not hesitated to conclude, that opportunities are often arising in the practice of surgery, wherein this operation would be not only justifiable, but as eligible and necessary as the operation operation for the radical cure of the Hydrocele. Under these impressions, I am ready to perform this operation whenever all the circumstances conspire to make it prudent, and to promise ultimate benefit to the patient. In your note to Correspondents, it appears that some person (perhaps interested to prevent the radical cure of ruptures) has endeavoured to condemn the attempt. Such person should wait until the arguments in its favor, and the plan to be adopted, are fully before the public; and which it is my intention to undertake as soon as the operation has been fairly tried. I am, &c.

*Soho Square, April* 14, 1803. A. CARLISLE.

---

